# Importance of the Ultrasonography in Diagnosis of Ileal Duplication Cyst

**DOI:** 10.1155/2013/248625

**Published:** 2013-11-04

**Authors:** Arzu Gebesce, Mevlit Korkmaz, Esengul Keles, Feride Korkmaz, Kamran Mahmutyazıcıoglu, Hamza Yazgan

**Affiliations:** ^1^Department of Pediatrics, Fatih University, Istanbul, Turkey; ^2^Department of Pediatrics Surgery, Fatih University, Istanbul, Turkey; ^3^Department of Anesthesiology, Fatih University, Istanbul, Turkey; ^4^Department of Radiology, Fatih University, Istanbul, Turkey

## Abstract

Gastrointestinal duplication cysts are rare congenital anomalies that can be seen in anywhere of the gastrointestinal system from the mouth to the anus. These are prenatally diagnosed through antenatal ultrasonography. However, attention must be paid since these formations might be confused with ovarian or mesenteric cysts. Our patient, who had been diagnosed with ovarian cyst on the ultrasonography performed in another center and with mesenteric cyst based on the abdominal MRI carried out at fifth month of life, presented to our clinic with the only complaint of constipation at 9th month of life. The diagnosis was set through double wall appearance of duplication cyst on the abdominal ultrasonography. The patient's cyst was resected.

## 1. Introduction

Gastrointestinal duplication cysts are rare congenital anomalies that can be seen in anywhere of the gastrointestinal system with a prevalence between 1 : 4500 and 1 : 10000. The most common location is ileum. Signs and symptoms differ based on the location, although mostly it manifests as acute abdominal or intestinal obstruction before 2 years of life. More rarely serious complications such as gastrointestinal bleeding or malignant degeneration may also occur. Colonic duplication cysts are further rare. Gastrointestinal duplication cysts are usually seen as two different forms as lumen-communicating and noncommunicating types. The most common form is cystic and noncommunicating type [1–3]. The treatment is surgery and total excision is the method of choice. In this report, our patient, who had been diagnosed with ovarian cyst on the ultrasonography (USG) performed in another center and with mesenteric cyst based on the abdominal MRI carried out at fifth month of life, presented to our clinic with the only complaint of constipation at 9th month of life. The diagnosis was set in our clinic through double wall appearance on the abdominal ultrasonography. The patient's cyst was resected.

## 2. Case Report

On the second-level USG ordered at 23rd gestational week of a healthy mother aged 32, a hypoechoic formation with 5 mm and 9 mm in size which was observed at the right side of the bladder in the pelvis was considered and followed up as ovarian cystic formation.

The patient was born by normal vaginal route at term, weight of 3000 gr. The patient's Apgar score was found 8 in minute 1 and 9 in minute 5. No pathologic finding was found at the physical examination of the patient. On the contrast enhanced abdominal MRI performed in a different center when the patient was five months old showed simple cyst. It also revealed two lobule-contoured, thick-walled cystic lesion with diameters of 19 mm, 12 mm, and 5 mm in the right lateral part of the abdomen. The mass was diagnosed as mesenteric cyst, and the patient was followed up for this diagnosis.

**Figure 1:**
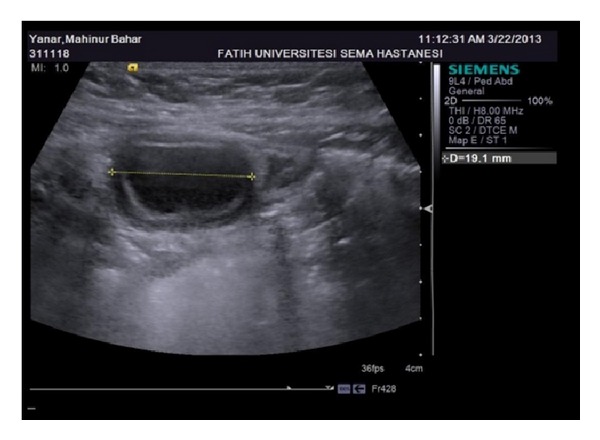
On sonography, we found that the hypoechoic outer wall of the cystic structure was continuous with the hypoechoic outer wall of the adjacent ileal bowel, and the hypoechoic outer wall of the cystic structure was continuous with the hypoechoic outer wall of the adjacent ileal bowel.

Our patient presented to our clinic at her 9th month of life with only complaint of constipation. On physical examination, biochemical and hematological values were found normal in the patient who had a growth and development of 50 percentile. On her USG performed in our clinic, 19 mm, 12 mm, and 5 mm in size the duplication cyst having double wall appearance was observed in the right lower quadrant. On sonography, we found that the hypoechoic outer wall of the cystic structure was continuous with the hypoechoic outer wall of the adjacent ileal bowel, and the hypoechoic outer wall of the cystic structure was continuous with the hypoechoic outer wall of the adjacent ileal bowel (Figure 1).

**Figure 2:**
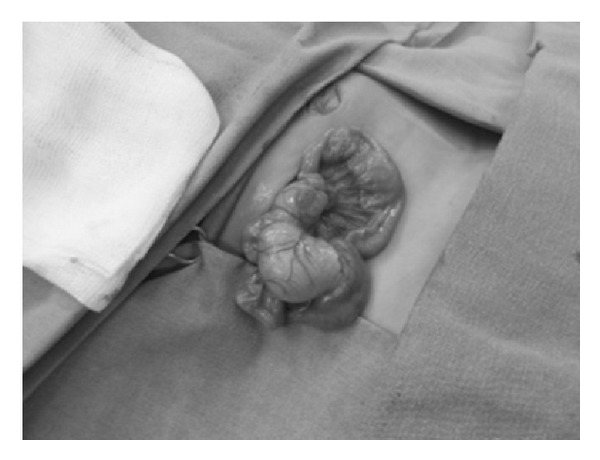
On exploration, a duplication cyst of 19 mm, 12 mm, and 5 mm in size was found at the ileal level at an approximate distance of 45 cm from the ileocecal valve.

An exploratory laparotomy was performed. On the exploration, a duplication cyst of 19 mm, 12 mm, and 5 mm in size was found at the ileal level at an approximate distance of 45 cm from the ileocecal valve (Figure 2). About 7 cm ileal segment involving the cyst was resected through distal and proximal bowel clamps. No complication was observed in postnatal followups of the patient. Histopathological examination of the piece was consisted with intestinal duplication cyst.

## 3. Discussion

Alimentary tract duplications are uncommon congenital anomalies that occur mostly in paediatric patients. The first reported case was made by Calder in 1733 [4]. Duplications arise from disturbances in the embryonic development of the gastrointestinal tract. Several major theories have been proposed for the formation of duplications at various sites, including the aberrant luminal recanalisation theory and the diverticular theory [5]. Others include the split notochord theory which explains the formation of neurenteric duplications and associated vertebral anomalies. Recently, Bishop and Koop suggested that environmental factors such as trauma or hypoxia in early foetal development were likely to be responsible when multiple duplications are found in association with anomalies such as malrotation or atresia [6].

Primary cystic lesions seen in the fetal and neonatal periods include renal, choledochal ovarian, mesenteric, duplication cysts, or Meckel's diverticulum. Double wall appearance of duplication is monitored on USG as a hyperechoic edge surrounding the mucosal wall inside and a hypoechoic wall surrounding the smooth muscular layer outside. However, the double wall sign can also be a characteristic of Meckel's diverticulum or sonographic artifacts. Furthermore, Meckel's diverticulum can have a spherically shaped cystlike appearance, which is similar to an enteric duplication cyst, on sonography [7–9]. Although Meckel's diverticulum is located on the antimesenteric border and communicates with the adjacent bowel lumen, in contrast to the duplication cyst, these differentiating features are often not visible on USG or CT [10, 11].

Presentation of intestinal duplications is variable depending on size, shape, and type of mucosa. They may be asymptomatic and discovered accidentally at surgery. They may be minimally symptomatic and associated with vague abdominal pain, constipation, or failure to thrive [12]. Our patient presented to our clinic at her 9th month of life with only complaint of constipation.

The enteric duplication cyst can be associated with malrotation. In half of the cases, there are associated malformations, the most frequent of these being esophageal duplications, followed by vertebral abnormalities [13]. No malrotation and malformations as esophageal duplications and vertebral abnormalities were seen in our case.

 Our patient, who had been diagnosed with ovarian cyst on the ultrasonography (USG) performed in another center and with mesenteric cyst based on the abdominal MRI carried out at fifth month of life, presented to our clinic with the only complaint of constipation at 9th month of life. On USG duplication cyst was diagnosed through the double wall appearance which is specific for duplication cyst. The treatment option proposed for duplication cysts is surgery and the surgery of choice is excision because of the potential complications [14]. In our patient also we performed surgical excision under general anesthesia.

## 4. Conclusion

In conclusion, ultrasonography is an inexpensive and practicable imaging modality in evaluation of intra-abdominal cystic lesions. Although it seems asymptomatic and rare, this type of cyst must be surgically excised because of the potential complications and malignant degeneration.
